# Japan

**Published:** 1882-03

**Authors:** 


### JAPAN.

RAPID PROGRESS MADE RECENTLY IN HIGHER CIVILIZATION.

The secretary of the American legation at Tokio, Japan, Mr. D. W. Stevens, lately arrived home after an absence of over eight years. He makes the following interesting statement to the Washington *Post:*

Japan is one of the most beautiful countries on earth, and a delightful place to live in. Since 1870 the progress of Japan has been rapid, and year after year its intercourse with foreigners has become more intimate and friendly. The Japanese manifest a great desire to become skilled in the arts and sciences of the more civilized nations. They are very apt scholars. All at once they undertook these great problems—to extinguish the feudal system, which included the whole nation, to reorganize their military system, to build railroads, telegraphs, lighthouses ; in fact, it is a difficult thing to imagine one great improvement that they did not attempt. In most of these undertakings they succeeded admirably. Take their educational problem. I have traveled on foot over most of the land, and I did not find a single village without its school-house, supplied with all the modern appliances for training the mind. Education is compulsory, and even the lowest can read and write. They have, by the way, magnificent normal schools for girls. They have an army of 35,000 well-equipped, and pronounced by military critics to be, No. 1 soldiers. They have, in my mind, only one fault, a slouchy way of marching, that they acquired from their teachers, the French. Their navy is on an equally good footing. Telegraph lines and submarine cables connect all the important points. They only have about 150 miles of railroads, but more lines are in contemplation. The commercial policy of the English is such as to command many privileges, but the Japanese undoubtedly consider Americans very friendly. They realize the fact that we are true friends, and have no inclination to force upon them our own wares at our own prices. They are daily becoming more and more independent, and as their education advances, they the more and more appreciate their importance and their rights under the laws of nations. They look upon America as their natural trading point, and if untrammelled, they would undoubtedly buy most of their necessities from us. The present ministry is exceedingly friendly to us. They are fond of the French; in fact, they are well disposed to all foreigners who treat them fairly and kindly. The time has passed by when the Japanese officers could be bullied into any measures they may deem detrimental.

Americans in Japan are treated with the utmost courtesy and attention and the greatest kindness. In the empire of Japan there are about 4,000 foreigners, and of these 800 are Americans. In Tokio, the capital, there are 200 Americans, and in Yokohama about 400. The others are scattered in the smaller cities. About 100 Americans are in the employ of the Japanese government and private Japanese companies. Americans have almost entire control of the educational affairs. By the way, the Japanese prefer America when they go abroad for an education. There are fully 300 American missionaries in Japan. They love the work, and they live pleasantly and with comparative ease. In Yokohama some of the large business houses are American. All the largest tea houses are American. The tea houses, by the way, have of late years sustained very heavy losses. America controls the kerosene trade, and this trade represents from 10,000,000 to 15,000,000 gallons annually. The United States is rapidly regaining its cotton trade with China, and in due time we are certain to secure the cotton trade in Japan. At present the English undertrade us by selling a very inferior article that, in external appearance, seems equal to the American article.

I should not advise any large number of Americans to go to Japan for business purposes, as the avenues of trade there are well taken up. Still a man with capital can make money in Japan in the not distant future. The Japanese aim to control their export trade, and even though trade with America may largely increase, Americans in Japan may not profit by the increase. Travelers, however, should not fail to visit Japan; it is, indeed, a lovely country, the epitome of beauty. The climate is very healthful. Tokio, for its size (850,000 people), is one of the healthiest cities in the world.

From an Oriental standpoint Japan is an extremely moral country. The condition of the women is excellent and steadily improving. Many of the women are engaged in business.

Mr. Stevens made a parting statement that will certainly be refreshing to American pride. Throughout Japan the name of Washington is a household word, and even the most ignorant seem aware of the fact that Washington was the first of Americans, and a great friend to humanity in general. No other foreign nation here was apparently known to the common people.

### THE RAREST OF GEMS.

The rarest of all gems is not the diamond, which follows after the ruby. This in its turn allows precedence to the chrysoberyl —popularly known as the cat's eye. The true stone comes from Ceylon, though Pliny knew of something similar under the name zimilampis, found in the bed of the Euphrates. Can we wonder, when we look at one of these singular productions of nature, with its silvern streak in the centre, and observe, as we move it ever so slightly, the magic rays of varying light that illumine its surface, that it was an object of profound reverence to the ancients? The possessor was supposed never to grow poorer, but always to increase his substance. The largest known is now in the possession of Mr. Bryce Wright, the well-known mineralogist. It is recorded in the annals of Ceylon, and known to history as the finest in the world. Two stars of lesser magnitude shine by its side, and we are informed that three such stones are not known to exist elsewhere in the wide world.—*London Graphic.*

Sanitary Errors.—1. To believe that the more hours children are at their studies the faster they learn. 2. To believe that the more a person eats the stronger and fatter he will become. 3. To believe that if exercise is good for one it should be taken at all hours and seasons—the more violent the better the result. 4. To imagine that the smallest room in the house is large enough to sleep in. 5. To eat without appetite. 6. To eat a hearty supper the last thing at night.

When the Old Testament is revised and modernized it will probably state that Adam, after eating the apple, received word that his resignation would be accepted.

### AFFECTATION.

Affectation is a source of discomfort, both to those who are guilty of it and to those who witness it. Nothing makes a person more ridiculous than conceit. Some people seem to think that a statesman must always be intensely stately, and a minister grave as a gravestone; the man of science must appear absorbed in some tremendous problem, and the poet must keep his eyes in a "fine frenzy rolling." If one thing more than another distinguishes a truly great man, it is his naturalness. What God made him, that he lives out. A man should be real, and in order to be so he must be himself. We should not trouble ourselves by thinking how we appear to observers. There is no greater folly than for people without brains to affect to be very wise, and people without money to affect to be very rich, and people without religion to affect to be great saints.

